# Supplementary material for: In Vitro and In Vivo Efficacy of a Stroma-Targeted, Tumor Microenvironment Responsive Oncolytic Adenovirus in Different Preclinical Models of Cancer
Source: Int J Mol Sci. 2023 Jun 10;24(12):9992. doi: 10.3390/ijms24129992 (PMC10297998; doi:10.3390/ijms24129992)
Supplement: Supplementary file 1 [file ijms-24-09992-s001.zip › Figure S1 25 de mayo 2023.pdf]

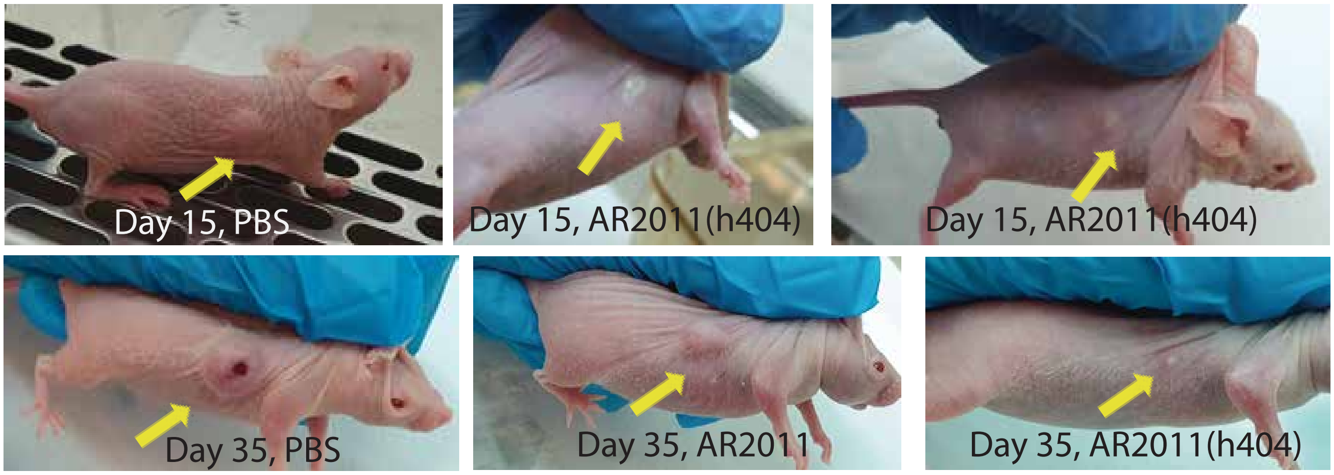

Figure S1: Photographs from in vivo studies in nude mice harboring s.c. tumors. Photographs were taken at different time points from mice harboring established tumors in the flank, and treated either with PBS, AR2011 or AR2011(404). Photographs were taken 15 and 35 days after the first administration either of the OAdVs or PBS. The arrows point to the tumor location.
