# Supplementary material for: In Vitro and In Vivo Efficacy of a Stroma-Targeted, Tumor Microenvironment Responsive Oncolytic Adenovirus in Different Preclinical Models of Cancer
Source: Int J Mol Sci. 2023 Jun 10;24(12):9992. doi: 10.3390/ijms24129992 (PMC10297998; doi:10.3390/ijms24129992)
Supplement: Supplementary file 1 [file ijms-24-09992-s001.zip › Figure S2 25 de mayo 2023.pdf]

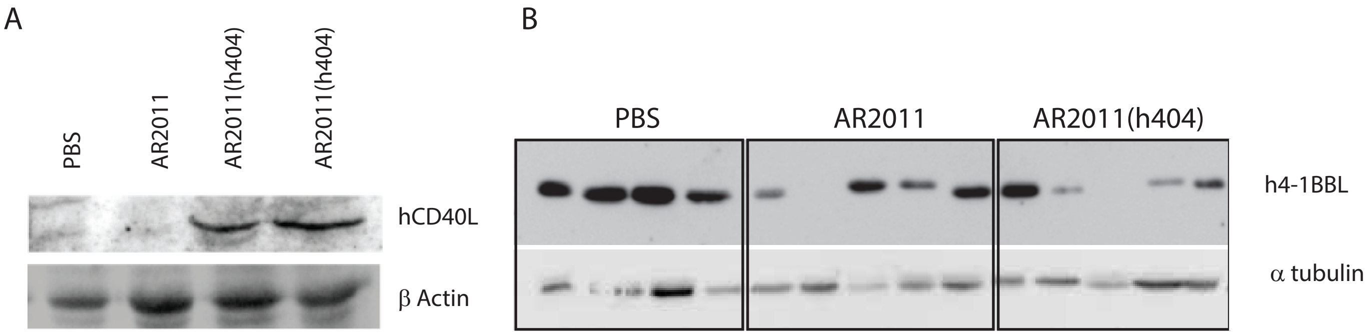

Figure S2. Western Blot of tumor samples obtained from in vivo studies in nude mice. Mice, 6-8 week old, were injected with $4.5x10^6$ SKOV-3 cells in the flank. When tumors reached 100 mm^3 in average, mice were administered i.t. with $5x10^{10}$ v.p. of PBS, AR2011 or AR2011(h404) once. Seventy-two hours later mice were sacrificed, the tumor area was removed and a protein extract was prepared for western blot analyses. The membranes were probed with anti-CD40L (A) and anti-h4-1BBL (B). Anti-β-actin and anti-α-Tubulin were used as a loading control.
