# Supplementary material for: In Vitro and In Vivo Efficacy of a Stroma-Targeted, Tumor Microenvironment Responsive Oncolytic Adenovirus in Different Preclinical Models of Cancer
Source: Int J Mol Sci. 2023 Jun 10;24(12):9992. doi: 10.3390/ijms24129992 (PMC10297998; doi:10.3390/ijms24129992)
Supplement: Supplementary file 1 [file ijms-24-09992-s001.zip › Figure S3 25 de mayo 2023.pdf]

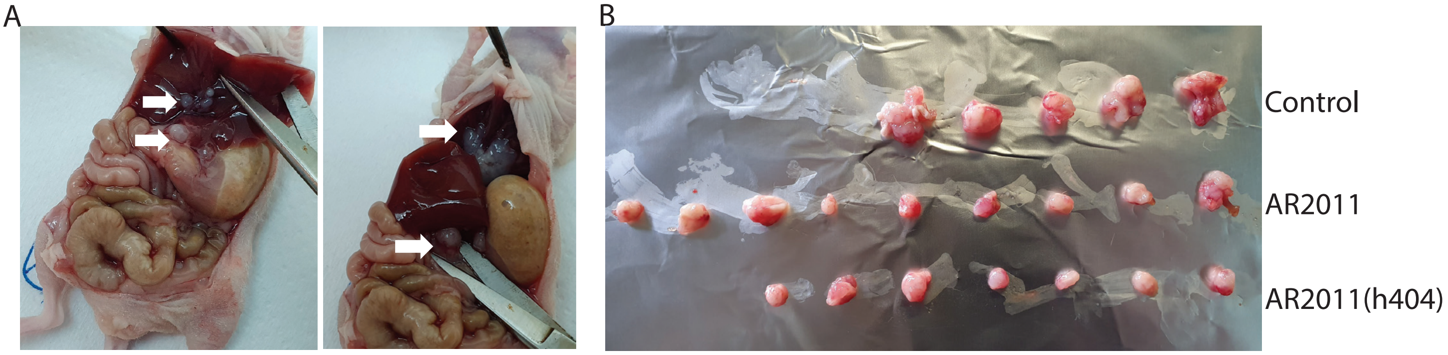

Figure S3. Photographs from in vivo studies in nude mice harboring intraperitoneal tumors. (**A**) Photographs were taken at autopsy on PBS-treated mice. The arrows show the location of metastatic nodules in the peritoneal cavity. (**B**) All the visible tumors were removed, collected for each single mouse and photographed.
