# Supplementary material for: In Vitro and In Vivo Efficacy of a Stroma-Targeted, Tumor Microenvironment Responsive Oncolytic Adenovirus in Different Preclinical Models of Cancer
Source: Int J Mol Sci. 2023 Jun 10;24(12):9992. doi: 10.3390/ijms24129992 (PMC10297998; doi:10.3390/ijms24129992)
Supplement: Supplementary file 1 [file ijms-24-09992-s001.zip › Figure S4 junio 2023.pdf]

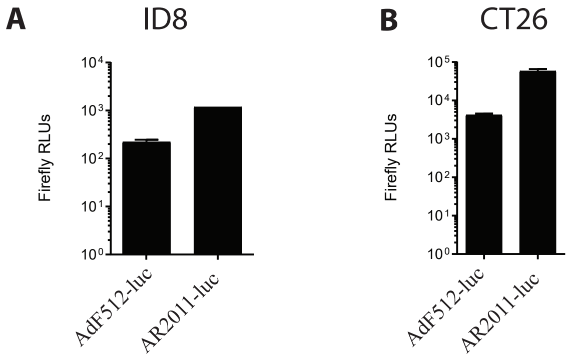

Figure S4. Transcriptional activity of the triple hybrid promoter in murine malignant cells. Luciferase activity of 2 versions of SPARC promoter, AdF512-luc [18] and AR2011-luc [43] in murine cells (A) ID8 cells and (B) CT26 cells. The bars represent mean±SD (n=2). CT26 and ID8 cells were plated into 24-well plates (50,000/well) 1 day before infection at MOI 500 of AdF512-luc or AR2011-luc in 200 μL of DMEM/F12 and 2% FBS. After 4-hr incubation, 800 μL of fresh medium was added and 72 hr later, the luciferase assay was assessed as described [18]. F512 is the SPARC promoter without the hypoxia and NFkB-responsive elements that are present in the AR2011 Promoter.
